# A rare case of conjunctival nevi involving the cornea

**DOI:** 10.11604/pamj.2023.44.167.39067

**Published:** 2023-04-12

**Authors:** Ankit Gupta, Sachin Daigavane

**Affiliations:** 1 Department of Ophthalmology, Jawaharlal Nehru Medical College and Acharya Vinoba Bhave Rural Hospital, Datta Meghe Institute of Higher Education and Research, Sawangi (M), Wardha, India

**Keywords:** Nevus, nevi, ophthalmology, pigmented axenfeld loop, malignant melanoma

## Image in medicine

Conjunctival melanocytic tumours comprise benign and malignant neoplasms. Nevi are congenital benign conjunctival melanocytic tumours. Primary acquired melanosis (PAM) can either be regarded as benign (PAM without atypia) or represent a precancerous lesion (PAM with atypia). In contrast, melanoma is, by definition, a malignant melanocytic tumour. This is a case of a 22-year-old female with a mass in her left eye (A). On slit lamp examination, the nevi were detected as shades of brown of size 5mm x 3mm located at the interpalpebral conjunctiva near the limbus involving the cornea at three o’clock, separated by normal conjunctiva. Increased blood supply and adherence to the underlying sclera were noted (B). Nevi can change colour and size during adolescence, but if a change is seen in an adult, a suspicion of malignancy should be ruled out. The malignant transformation risk is 1%. Inspection of all ocular structures was done as multicentric lesions could be present. It was excised and submitted for a histopathological investigation that confirmed the diagnosis (C). There is a social stigma about conjunctival nevus as it is cosmetically appealing. Surgical excision with an amniotic membrane graft or mitomycin C or conjunctival autograft is the mode of treatment, though the corneal opacity cannot be reversed.

**Figure 1:**
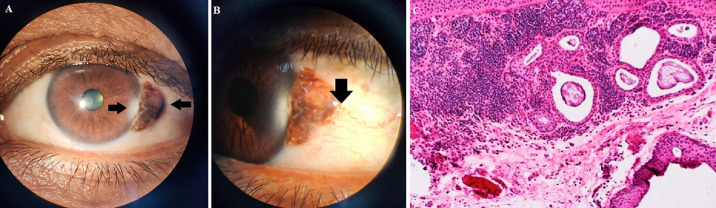
A) front gaze view of the nevi showing the corneal involvement and brown coloured nevi at 3 o’clock; B) side gaze view of the nevi showing increased vasculature; C) nests and diffuse collections of subepithelial nevus cells showing the entrapped epithelial inclusion cysts and the total absence of intraepithelial nevocytic junctional nests (hematoxylin-eosin, original magnification x100)

